# Collagen concentration and biomechanical properties of samples from the lower uterine cervix in relation to age and parity in non-pregnant women

**DOI:** 10.1186/1477-7827-8-82

**Published:** 2010-07-06

**Authors:** Birgitte S Oxlund, Gitte Ørtoft, Annemarie Brüel, Carl Christian Danielsen, Pinar Bor, Hans Oxlund, Niels Uldbjerg

**Affiliations:** 1 Department of Obstetrics and Gynecology, Aarhus University Hospital, Skejby, DK-8200 Aarhus N, Denmark; 2 Institute of Anatomy, Aarhus University, DK-8000 Aarhus C, Denmark; 3 Department of Obstetrics and Gynecology, Regional Hospital of Randers and Grenaa, DK-8930 Randers, Denmark

## Abstract

**Background:**

During normal pregnancy the cervix has a load bearing function. The cervical tissue consists mainly of an extracellular matrix (ECM) rich in collagen; important for the biomechanical properties. The aim of the present study was to evaluate how the biomechanical strength of samples from the distal cervix is associated with collagen content in relation to age and parity. This study demonstrates a method to investigate cervical tissue from women who still have their uterus in situ.

**Methods:**

Cervical punch biopsies (2 × 15 mm) were obtained from 57 healthy women (median age: 39 years, range: 29-49 years). Biomechanical tensile testing was performed, and collagen concentration (as % of dry defatted weight (DDW)) and content (mg of collagen per mm of specimen length) was determined. Histomorphometry was used to determine the volume densities of extracellular matrix and smooth muscle cells. Smooth muscle cells were identified by immunohistochemistry. Finally, orientation of collagen fibers was estimated. Data are given as mean +/- SD.

**Results:**

The mean collagen concentration (62.2 +/- 6.6%) increased with age (0.5% per year, r = 0.45, p = 0.003) and decreased with parity (1.7% per birth, r = -0.45, p = 0.033). Maximum load was positively correlated with collagen content (mg of collagen per mm of specimen length) (r = 0.76, p < 0.001). Normalized maximum stiffness was increased with age (r = 0.32, p = 0.017), whereas no correlation was found with regard to parity. In tissue samples with a length of approximately one cm, volume density of smooth muscle cells increased gradually from 8.9% in the distal part near the epithelium, to 15.5% in the proximal part (p < 0.001).

**Conclusions:**

The present study shows that cervical collagen concentration increases with age and decreases with parity in non-pregnant women. In addition, collagen stiffness increased with age, whereas no change in collagen tensile strength with respect to age and parity was found. These results show that collagen contributes to cervical tissue tensile strength and age and parity should be considered confounding factors.

## Background

During normal gestation the uterine cervix has a load bearing function. It needs to withstand the pressure from the growing fetus and not dilate during pregnancy. At birth the cervix softens and ripens to allow delivery of the fetus. These biomechanical properties of the cervix should most likely be described in terms of connective tissue biology as the cervix contains less than 15% smooth muscle cells, the rest being dominated by an extracellular matrix (ECM) rich in collagen [1]. The biomechanical strength of connective tissue is, however, determined not only by the collagen concentration, but also by the distribution of collagen types (predominantly types I and III, IV) [2,3], the proteoglycans decorin and biglycan which affect collagen fibrillogenesis [4,5], the amount and types of collagen cross-links [6,7], the orientation of collagen fibers [8,9] and the concentration of elastin and water [10].

Age-related changes in the cervix are not well described. Investigations on connective tissue and aging derive predominantly from studies on bone, cartilage and muscle [11]. In most connective tissues there will be a decrease in the amount of tissue due to an imbalance in matrix synthesis and breakdown [11]. It is suggested that cervical collagen concentration is increased with age whereas water content is decreased [12].

During pregnancy changes in cervical tissue occur as the consistency of the cervix changes. Collagen concentration is decreased whereas collagen extractability and water content are increased during pregnancy and labor [13,14]. It is, however, unknown whether collagen concentration (or ECM) and mechanical strength of cervical tissue reach previous levels before pregnancy. Studies indicate that the collagen concentration is decreased with number of births [15]; however, it is unknown whether collagen is replaced by smooth muscle cells and whether orientation of collagen fibers is the same after pregnancy.

The aim of the present study was to investigate the relationship between collagen content and biomechanical strength in the uterine cervix. We also aimed to examine the effect of age and parity on mechanical strength, collagen concentration and orientation as an understanding of these relationships may be important in investigating a dysfunctional cervix. We want to use the method for investigation of cervical tissue from women with cervical insufficiency. The study is one of the few which describes a biomechanical method to investigate cervical biopsies from women in the fertile age who still have their uterus in situ.

## Methods

### Study population

Fifty-seven healthy non-pregnant women (median age: 39 years, range: 29-49 years, median parity: 2, number of nullipara: 5), admitted for sterilization, were included in the study. Exclusion criteria: history of preterm delivery, conization, cervical laceration, cervical dysplasia, menopause or connective tissue disorders. The study was approved by the Local Research Ethical Committee (Region of Midtjylland, journal number: 20040195) and conducted in accordance with the Declaration of Helsinki 2008. Written informed consent was obtained from each woman who participated in the study.

### Tissue collection

Long, narrow biopsies of cervical tissue (approximately 15 × 2 mm) were punched out parallel to the cervical canal, halfway between the external os and the lateral surface of the cervix, with an instrument of external diameter 3 mm (Miltex^®^, Dermal Biopsy Punch, Germany). Three biopsies were obtained from each patient at the 3, 6 and 12 o'clock positions. Hemostasis was secured by compression, or if necessary with electrocoagulation or a stitch. No complications were observed apart from slight vaginal bleeding.

Two biopsies were immersed in Ringer's solution and immediately frozen at -80°C until biomechanical testing. The third biopsy was divided in a proximal and a distal portion. The proximal portion, approximately 5 mm long, was used for later genetic studies. The distal part including the epithelium, approximately 10 mm long, was immersion fixed in 0.1 M sodium phosphate-buffered 4% formaldehyde, pH 7.0, for 24 hours and stored in 70% ethanol until histological examination.

### Biomechanical analysis

Two biopsies from each patient were analyzed by means of a materials testing machine (Alwetron TCT5, Lorentzen & Wettre, Kista, Sweden). The biopsies were thawed at room temperature, and the epithelium removed using a dissecting microscope. Each sample, immersed in Ringers solution (pH 7.4), was mounted between two clamps with a jaw space of 4 mm. The tensile strength of specimens was tested by moving the clamps apart with a constant deformation rate (10 mm/min), stretching the sample until breaking, while a load-deformation curve was recorded. Subsequently, the tissue between the clamps was isolated, and used for determination of hydroxyproline.

**Figure 1:**
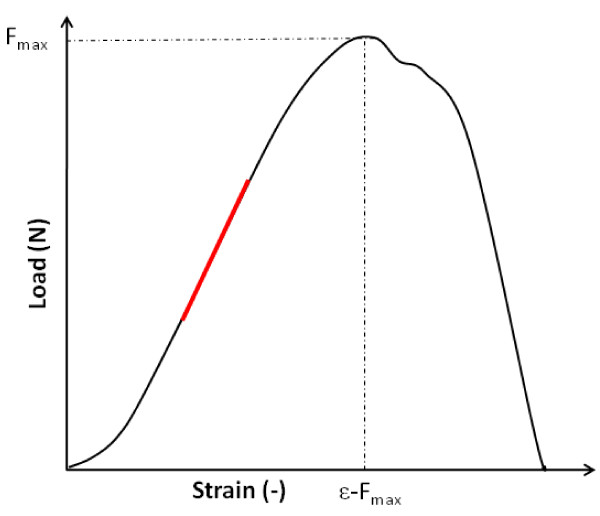
**A typical load-strain curve for cervical tissue samples**. The diagram illustrates the biomechanical parameters; F_max _(maximum load), *ε*--F_max _(maximum extensibility) and maximum stiffness (red bar = maximal slope of load-strain curve).

From the load-strain curve (Figure 1) the following parameters were derived:

F_max _: maximum load (N); the maximum force used for breaking the specimen.

ε-F_max_: strain at maximum load; the ultimate specimen extensibility.

S_max_: maximum load; normalized for collagen (N × mm × mg^-1^).

S'_max_: maximum stiffness; normalized for collagen (N × mm × mg^-1^).

F_max _and ε-F_max _imply biomechanical characteristics of the specimen, whereas S_max _and S'_max _imply characteristics of the collagen component.

### Determination of hydroxyproline

After the biomechanical analysis, the tissue between the clamps was used for hydroxyproline analysis. The tissue was defatted in acetone and after freeze-drying the DDW was determined. The tissue was then hydrolyzed in 6 M HCl for 16 h at 100°C. Subsequently the hydroxyproline content was measured according to Woesssner [16] with modifications as described [17]. The collagen content was calculated by multiplying the hydroxyproline content by 7.46 [18].

### Immunohistochemistry

Immunohistochemistry was used to detect smooth muscle cells. The tissue biopsies were embedded in paraffin and 2-μm-thick longitudinally sections were cut and mounted with two sections per slide. The sections were deparaffinized, and endogenous peroxidase was blocked by 0.5% H_2_O_2 _in absolute methanol. In order to reveal antigens, the sections were boiled for 10 min. in 0.1 mM Tris/HCl and 0.5 mM EGTA, pH 9. Non-specific binding was blocked by 1% BSA (bovine serum albumin). The sections were incubated overnight at 4°C with a primary antibody against smooth muscle actin (1:1600, monoclonal mouse anti-human, M0851, DAKO, Denmark) diluted in PBS supplemented with 0.1% BSA and 0.3% Triton-X100. Negative controls were incubated with mouse serum or IgG1 instead of primary antibody. After washing, the sections were incubated with horseradish peroxidase-conjugated secondary antibody (goat anti-mouse P0447, DAKO, Denmark), for 1 h at 20°C. The peroxidase was visualized by reaction with 0.05% 3,3'-diaminobenzidine tetrahydrochloride dissolved in PBS with 0.1% H_2_O_2_, before counterstaining with Mayer's Haematoxylin and alcoholic eosin. Sections with muscular arteries were used as positive controls.

### Estimation of the volume density of extracellular matrix (ECM) and muscle cells

For histomorphometry a modified Olympus BH-2 microscope with a motorized stage was used combined with a charge coupled device video camera (JAI-2040, Kanagawa, Japan) under computerized control. By means of CAST software (Olympus, Denmark), counting frames were superimposed onto live images of the tissue sections. The fields of view in each section were sampled using systematic, uniformly random sampling (SURS) [19]. From a random starting point, a new field of view with a fixed x and y distance from the previous field was sampled by means of a motorized specimen stage. For each patient two immunostained sections were evaluated. The epithelium was used to determine the original orientation of the sample. Each section was divided into three or four 2 mm sites, depending on biopsy length (i.e. sites I-IV: 0-2, 2-4, 4-6, 6-8 mm from the epithelium, respectively). Only sections with detectable epithelium were included in this analysis (n = 50). In each section approximately 36 fields of view were evaluated using a counting grid of 81 points. The number of points hitting ECM (defined as non-cellular components), smooth muscle cells (positive for smooth muscle actin), nuclei associated with the connective tissue and blood vessels (with a visible lumen, vessel wall and eventually blood cells) were counted. Counting took place at a final magnification of × 1263. Evaluation of sections was blinded.

### Determination of collagen orientation

Collagen orientation was determined by microscopy (Olympus BX40) with live video imaging (Nikon DS-Fi1), connected to a monitor (Sony Multiscan G200). Nikon NIS-Elements F 3.00 software was used. Three μm thick sections were cut parallel to the long axis of the biopsy, and stained with Picro-Sirius [20]. The sections were divided into two portions, of which the proximal portion, corresponding to the part used for mechanical analysis, was used for determination of collagen orientation. The epithelium was used to determine section orientation. Only sections with detectable epithelium were included (n = 42). A grid was constructed and physically mounted on the computer monitor. With the longitudinal axis of sections horizontal, collagen fibers at least 27 μm long were divided into three categories based on their orientation: 1) fibers deviating less than ± 45° from the longitudinal axis; 2) fibers deviating between 46° and 90° or -46° to -90° from the longitudinal axis; 3) fibers shorter than 27 μm long (indicating that they are not oriented parallel with the sectioning plane). The ratio of fibers "parallel" with the longitudinal axis (category 1) to total collagen (category 1 + category 2 + category 3) was calculated. Two sections from each patient were evaluated at a total magnification of × 1115. In each section, collagen orientation was determined at four points within each of 25 randomly (manually) selected fields of view. As above, evaluation of sections was blinded.

### Statistical analyses

Data are given as mean ± SD. Linear regression was performed to describe the relationship between a pair of parameters. Multiple linear regression was used to adjust parameters for age and parity. If necessary data were log-transformed to comply with the assumption of the statistical method.

Repeated Measures ANOVA was performed to compare two different sites of the histological sections, whereas Test for trend was used to describe a gradual increase or decrease throughout the biopsy. Differences were considered significant when p < 0.05. Sigma stat 3.5 and STATA, intercooled 9 were used as statistical packages.

## Results

### Collagen and biomechanical analyses

**Figure 2:**
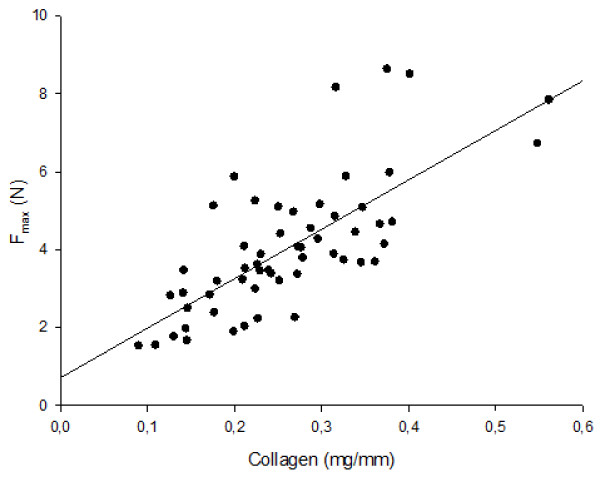
**Maximum load versus collagen content**. The maximum load (F_max_) of cervical samples in relation (linear regression) to unit collagen (mg of collagen per mm of specimen length) (r = 0.76, p = 0.001).

The biochemical and biomechanical values are shown in Table 1. Maximum load (tensile strength) was directly proportional to collagen content, given in mg per mm of specimen length (r = 0.76, p = 0.001) (Figure 2).

**Table 1:** Biochemical and biomechanical data

	Non-pregnant women
Number of individuals	56
Collagen % (mg/mg DDW)	62.2 ± 6.6
Collagen (mg/mm)	0.26 ± 0.01
F_max_, Maximal load (N)	4.0 ± 1.7
S_max_, Normalized maximal load (N × mm × mg^-1^)	15.8 ± 4.4
S_max_, Normalized maximal stiffness (N × mm × mg^-1^)	64.4 ± 21.9
ε-F_max_, Extensibility	0.49 ± 0.1

Cervical samples from non-pregnant women. Mean ± SD

### Histomorphometry

**Figure 3:**
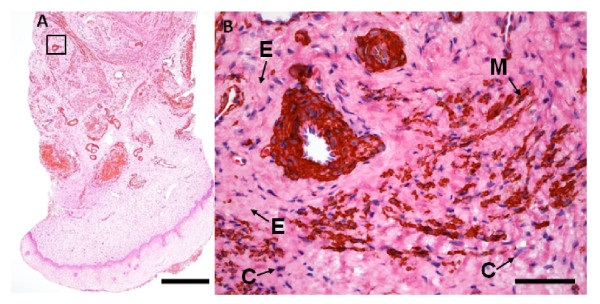
**Immunohistochemical staining of smooth muscle actin in section from the human cervix**. (A) Longitudinal section of a biopsy including the epithelium. Bar: 500 μm, (B) a blood vessel surrounded by extracellular matrix (E), smooth muscle cells (M) and connective tissue nuclei (C) (details from boxed-region in A). Bar: 50 μm. Sections were immunostained with an antibody against smooth muscle actin and counterstained with HE.

Sections from 57 women were examined by histomorhometry and smooth muscle actin was distinguished from ECM as shown in Figure 3. Mean volume density, as estimated by histomorphometry, was ECM 76.7 ± 7.2%, muscle cells 13.0 ± 6.6%, connective tissue nuclei 4.4 ± 1.4%, and blood vessels 5.9 ± 3.3%.

Although the punch biopsies were only 10 mm long, differences were observed between the proximal and distal ends (Table 2). Volume density of smooth muscle cells increased gradually from the distal part near the epithelium to the proximal part. The inverse was demonstrated by ECM volume density and ratio of ECM to muscle cells. When comparing the most distal site (I) with the most proximal (IV) within biopsies, a statistically significant increase in volume density of smooth muscle cells was found (p < 0.001) as well as a decrease in the ECM/muscle ratio (p < 0.001).

**Table 2:** Histomorphometry

	Site I	Site II	Site III	Site IV
	0-2 mm from the epithelium	2-4 mm from the epithelium	4-6 mm from the epithelium	6-8 mm from the epithelium
ECM %	78.7 ± 8.3	76.0 ± 7.4	76.5 ± 7.2	75.7 ± 4.6^†^
Muscle %	8.9 ± 6.7	13.5 ± 7.1	13.8 ± 6.3	15.5 ± 5.3**.^††^
Connective tissue nuclei %	6.4 ± 2.9	3.8 ± 1.5	2.9 ± 1.1	2.9 ± 1.4**,^††^
Blood vessels %	6.0 ± 3.6	6.8 ± 4.3	6.7 ± 4.7	5.9 ± 4.2
ECM/muscle ratio	18.9 ± 20.3	8.4 ± 6.7	7.8 ± 7.8	5.7 ± 2.7**,^††^

Histological sections of cervical biopsies from non-pregnant women. Mean ± SD

*p > 0.05 against site I by Repeated Measures ANOVA

**p < 0.001 against site I by Repeated Measures ANOVA

^† ^p < 0.05 comparison of site I-IV by test for trend

^††^0 < 0.001 comparison of site I-IV by test for trend

Relationships between histomorphometry and biomechanical strength were examined. Normalized maximum load (S_max_) (r = 0.43, p = 0.006) and normalized maximum stiffness (S'_max_) (r = 0.47, p = 0.002) were positively correlated with ECM volume density when data were adjusted for age.

### Collagen orientation

**Figure 4:**
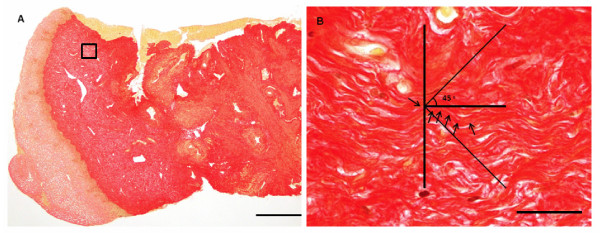
**Collagen fiber orientation**. Picro-Sirius stained sections from the human cervix. (**A**) Longitudinal section of a biopsy including epithelium (Bar: 500 μm), (**B**) collagen fibers in the center of the grid was divided into three categories based on their orientation (longitudinal axis of sections horizontal): **1**) longitudinal or "parallel" fibers (deviating less than ± 45° from the longitudinal axis), **2**) perpendicular fibers, (deviating between 46° and 90° or -46° to -90° from the longitudinal axis) representing circular or radial fibers, **3**) fibers shorter than 27 μm (not oriented parallel with the sectioning plane) representing circular, radial or wavy longitudinal fibers (arrows point at a longitudinal collagen fiber. Bar: 25 μm).

The percentage of collagen considered "parallel" (within 45°) with the longitudinal axis of the biopsies was 36.8 ± 9.6% (Figure 4), whereas the collagen considered "perpendicular" (greater than 45°) to the axis was 22.4 ± 9.7%. The remaining 40.6 ± 11.6% was not oriented in the plane of sectioning. No relationship between collagen orientation and normalized maximum strength (S_max_) was demonstrated (parallel fibers, r = 0.26, p = 0.098).

### Age

**Figure 5:**
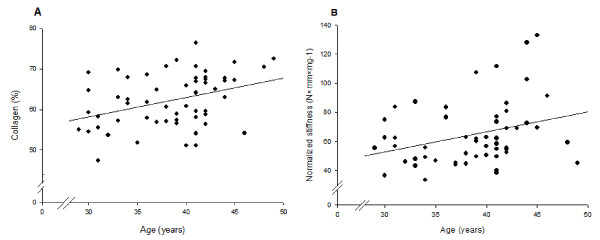
**Age in relation to collagen concentration and normalized maximum stiffness**. Age in relation (linear regression) to **(A) **collagen concentration (mg collagen/mg DDW × 100) of cervical samples from non-pregnant women (unadjusted by simple regression: r = 0.36, p = 0.006, adjusted for parity by multiple linear regression: r = 0.45, p = 0.003) and **(B) **normalized maximum stiffness (unadjusted: r = 0.32, p = 0.017, adjusted for parity: r = 0.33, p = 0.017).

Collagen concentration increased 0.5% per year of age (r = 0.45, p = 0.003), after adjustment for parity by multiple linear regression (Figure 5). Normalized maximum load showed no correlation to age, whereas, normalized maximum stiffness was increased with age.

The ECM volume density increased 0.5% per year, (adjusted for parity, r = 0.35, p = 0.017); however, no relationship was found between age and smooth muscle cell volume density (r = 0.17, p = 0.34). Also no relationship between collagen orientation and age was demonstrated.

### Parity

**Figure 6:**
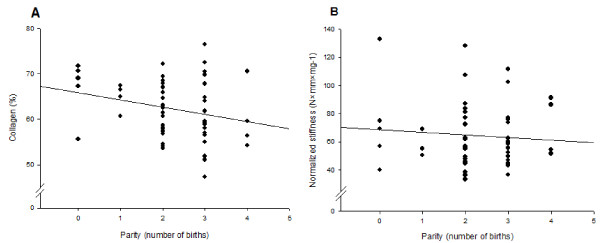
**Parity in relation to collagen concentration and normalized maximum stiffness**. Parity in relation (linear regression) to **(A) **collagen concentration (unadjusted: r = -0.25, p = 0.068, adjusted for age: (r = -0.45, p = 0.033) and (B) normalized maximum stiffness of cervical samples (unadjusted: r = -0.06, p = 0.67, adjusted for age: r = -0.33, p = 0.55).

The effect of parity was also evaluated. Collagen concentration decreased 1.7% per birth (r = -0.45, p = 0.033), when data were adjusted for age by multiple linear regression (Figure 6).

Normalized maximum load and stiffness showed no correlation to parity. No correlation was found between volume densities of ECM (adjusted for age, r = 0.35, p = 0.23) or smooth muscle cells (r = 0.17, p = 0.43) and parity. A significant correlation between parity and collagen fibers oriented parallel with the section (r = -0.35, p = 0.022) was found. No correlation between parity and perpendicular fibers or fibers not oriented in the plane of sectioning was shown.

## Discussion

The present study shows that ECM including collagen contributes to mechanical strength of cervical tissue whereas muscle cells seem to add sparsely. We had expected that the orientation of collagen fibers would affect the mechanical strength; however, we did not find any relationship between biomechanical strength and direction of collagen fibers. We found the collagen concentration increased with age and decreased with parity; whereas, no similar change was seen with respect to muscle cells. We also demonstrated a decrease in collagen fibers oriented in the longitudinal direction of the cervix with increasing parity.

Our results confirm previous studies showing that the biomechanical properties of cervical tissue are derived primarily from ECM in a non-linear stress-strain behavior [21-23]. Collagen is an important constituent of ECM and our study shows that collagen contributes to mechanical strength and muscle cells seem to contribute less [21]. Collagen cross-links, collagen fiber orientation as well as proteoglycans might be important contributors to the mechanical quality of the collagen.

The direction of collagen fiber orientation determines the direction in which the tissue best can withstand tensile forces [8]. In the present study a rough estimate of the collagen fiber orientation was obtained, and no predominant direction was observed. These data may therefore explain mechanical data from a previous study reporting similar tensile strength of longitudinal and circular cervical samples [21]. In the present study approximately 40% of the collagen fibers were oriented "parallel" with the mechanical testing direction, suggesting that these collagen fibers are main responsible for the tensile strength obtained during the mechanical testing of the biopsies. The method used for determination of collagen fiber orientation is rough but allows for detection of major differences. A comprehensive investigation of the cervical collagen orientation would require serial sectioning and a detailed 3-dimensional reconstruction.

We studied biopsies from the lower 10 to 12 mm of the cervix and found that, even within this small distance, muscle cell concentration varied with biopsy depth. The relative content of smooth muscle cells increased from 9% at a distance of 0 to 2 mm from the epithelium to 16% at a distance of 6 to 8 mm from the epithelium. Our results are in accordance with previous findings by Danforth, who demonstrated that the cervix predominantly consists of fibrous connective tissue with an average of only 15% smooth muscle cells [1], and Rorie and Newton who showed that smooth muscle cell concentration in the lower cervix was 6%, 29% in the upper part of the cervix, and further increased to 69% in the myometrium [24]. This is an important information for future clinical studies on relationship between cervical connective tissue and cervical dysfunction for example during pregnancy.

In agreement with Roberts *et al. *[12] the present study indicates that cervical collagen concentration increases with age. As high collagen concentrations are associated with long duration of labor [13], this corresponds well to studies showing that dystocia is more common among older women [25]. In contrast, Petersen *et al. *investigated 31 premenopausal women (age 19-45), but did not find any association between cervical collagen and age among nulligravidae [15]. The fact that only 5 nulliparous women were included in the present study could be responsible for the differences in these results. Studies on related tissues as the oviduct has shown similar increase in collagen concentration with age [26] and studies on skin has demonstrated an increase in collagen cross-linking with age [27]. The increase in collagen concentration with age demonstrated in the present study was supported by an increase in ECM found by histomorphometry. This age-related increase in ECM was not accompanied by a decrease in smooth muscle cells. Additionally, normalized maximum stiffness increased with age but not with parity. Therefore it could be suggested that the increase in ECM volume density is responsible for the increased stiffness. Other studies on connective tissue have shown similar age-related increase in stiffness [11].

In agreement with the above data, Petersen *et al. *demonstrated that collagen concentration decreases with increased parity. This inverse relationship between parity and collagen concentration might explain why parous women have lower frequencies of prolonged labor [28], supporting the hypothesis that composition of the non-pregnant cervix might determine the duration of labor at term, and perhaps the risk of preterm labor and cervical insufficiency. During pregnancy the collagen organization of cervical tissue decreases [29]. However, we found a decrease in longitudinal fibers with increasing parity, which may suggest a change in organization of the collagen fibers after pregnancy.

A major strength of the present study is the combined application of biomechanical, histomorphologic and biochemical analyses. Several studies use hysterectomy specimens for mechanical testing [21,22]; however we demonstrate a method to obtain longitudinal cervical biopsies suitable for mechanical testing. This enables biopsies from younger women with no known cervical diseases with their uterus in situ. We plan to apply these biomechanical, histomorphologic and biochemical analyses on cervical tissue from women with cervical insufficiency. It is assumed that cervical insufficiency results from the cervix being unable to resist normal passive load [30]. Petersen *et al. *have shown that non-pregnant women with a history of CI have a decreased cervical collagen concentration [15], and Warren *et al. *have demonstrated polymorphisms in the collagen 1A1 gene (*COL1A1*) in patients with CI [31]. Furthermore, Buckingham *et al. *have proposed that a muscular cervix, with abundance of muscle cells, may cause CI [32,33]. This supports a hypothesis about a possible constitutional defect of the cervical tissue, which is present also in a non-pregnant state.

In the present study we examined women with normal obstetrical history. Future studies are needed to evaluate whether women with cervical insufficiency have pathological changes i.e. collagen cross-links, proteoglycans or collagen orientation in addition to suggested changes in collagen concentration [15].

## Conclusions

The present study shows that in women with normal obstetrical histories, cervical collagen concentration increased with age and decreased with parity. This age-related increase in collagen was not accompanied by a decrease in smooth muscle cells. We found no change in the tensile strength of collagen (normalized maximum load) with respect to age or parity, however, the collagen stiffness increased with age. These results show that collagen contributes to biomechanical strength of the cervical tissue and age and parity should be considered confounding factors.

## Competing interests

The authors declare that they have no competing interests.

## Authors' contributions

BO, GØ, AB, HO, CCD and NU contributed to the design of the study. The sample collection was carried out by BO, GØ and PB. Biomechanical and histological analyses were done by BO, AB and CCD. BO, GØ, AB, CCD and NU participated in the data analyses. The manuscript was written by BO with revision by NU, GØ, AB, CCD, HO and PB. All authors have read and approved the final manuscript.
